# The Effect of Individualized Fall Prevention Programs on Community-Dwelling Older Adults: A Scoping Review

**DOI:** 10.7759/cureus.23713

**Published:** 2022-03-31

**Authors:** Lori E Boright, Sara K Arena, Christopher M Wilson, Lauren McCloy

**Affiliations:** 1 Physical Therapy, Oakland University, Rochester, USA; 2 Physical Therapy, Team Rehabilitation, St. Clair Shores, USA

**Keywords:** geriatrics, physical therapy, rehabilitation, exercise, comprehensive geriatric assessment, independent living, community-dwelling, falls, older adult, prevention

## Abstract

An alarming rate of injurious falls among older adults warrants proactive measures to reduce falls and fall risk. The purpose of this article was to examine and synthesize the literature as it relates to programmatic components and clinical outcomes of individualized fall prevention programs on community-dwelling older adults. A literature search of four databases was performed using search strategies and terms unique to each database. Title, abstract, and full article reviews were performed to assure inclusion and exclusion criteria were met. Data were analyzed for type of study, program providers, interventions and strategies used to deliver the program, assessments used, and statistically significant outcomes. Queries resulted in 410 articles and 32 met all inclusion criteria (19 controlled trials and 13 quasi-experimental). Physical therapists were part of the provider team in 23 (72%) studies and the only provider in 10 (31%). There was substantial heterogeneity in procedures and outcome measures. Most common procedures were balance assessments (n=30), individualized balance exercises (n=29), cognition (n=21), home and vision assessments (n=16), specific educational modules (n=15), referrals to other providers/community programs (n=8), and motivational interviewing (n=7). Frequency of falls improved for eight of 13 (61.5%) controlled trials and four of five (80%) quasi-experimental studies. Balance and function improved in six of 11 (54.5%) controlled trials and in each of the six (100%) quasi-experimental studies. Strength improved in three of seven (43%) controlled trials and four of five (75%) quasi-experimental studies. While many programs improved falls and balance of older adults, there was no conclusive evidence as to which assessments and interventions were optimal to deliver as individualized fall prevention programming. The skill of a physical therapist and measures of fall frequency, balance, and function were common among the majority of studies reviewed. Despite the variability among programs, there is emerging evidence that individualized, multimodal fall prevention programs may improve fall risk of community-dwelling older adults and convenient access to these programs should be emphasized.

## Introduction and background

Falls are defined as a person coming to rest inadvertently on the ground, floor, or other lower level [1,2]. The physical outcomes of each fall event may range in severity from no injury to death. While there may be no visible injury, increased fear of falling and decreased confidence when performing activities of daily living may increase future fall risk [3,4]. The United States (US) Center for Disease Control and Prevention (CDC) reports falls as the leading cause of injury deaths among those over 65 years [5]. Furthermore, the medical costs of an emergency department visit after a fall average 3038 United States Dollars (USD) and increase to 38,412 USD if the individual requires hospitalization [5]. The alarming rate of falls among older adults in combination with reports that 38% of these falls will require medical treatment warrants proactive measures to reduce falls and fall risk in this population [6].

Preventative, or upstreaming, approaches to decreasing falls are likely to reduce the associated downstream cost and personal burden. Furthermore, when positive or improved health outcomes are achieved at a reduced cost, the value of the service is improved to the benefit of both the healthcare system and the patient [7]. Common upstream strategies used to reduce fall burden include targeted education and balance and strengthening exercises. These intervention modes can be delivered in a group or individualized setting and by instructors with various backgrounds including public health, fitness, or healthcare. There are numerous tools to assess fall risk and it is important to determine what combination of tools and interventions are most clinically advantageous, as not all fall prevention strategies are useful for all individuals [8,9]. Given the complexity of variables impacting fall risk, individualized assessment and programming delivered by persons with skills that reach across both the public health and healthcare domains may be valuable.

There are a wide variety of interventions, programs, and options for fall prevention, but it is possible that the heterogeneity may limit systematic applicability for addressing falls. The CDC suggests that the Otago Exercise Program (OEP) and Stepping On program may be effective and have the potential for a strong return on investment as community-based fall prevention strategies [10,11]. The OEP was first implemented in New Zealand and uses a physical therapist (PT)-led individualized exercise program that incorporates muscle strengthening, balance retraining, and a walking program delivered using seven home visits and seven telerehabilitation visits over a 12-month time frame [11,12]. The OEP uses exercise as its primary intervention strategy for fall reduction and providers of this program must complete the required training prior to administration. The Stepping On program was first introduced in Australia by an occupational therapist (OT) [11]. The US-based version incorporates two-hour community-based group sessions conducted over seven weeks by trained leaders [11,13]. The educational topics are multifactorial, but the in-home sessions are self-guided by the learner.

Many fall-prevention programs are either in a group format in a community setting or require traditional healthcare services (e.g., outpatient physical therapy). If a community-dwelling older adult has a non-injurious fall or demonstrates a functional decline, it may impede their ability or confidence to leave their home to participate in group exercise programming or access outpatient physical therapy services. The inability to participate in community-based programming can also be compounded if transportation options are reduced, or during inclement weather. However, this leaves a gap in the services available to this older adults as they do not yet meet the Medicare definition of homebound but are having difficulty safely moving within the context of a community [14]. Additionally, falls most commonly occur in the home resulting from many factors beyond the physical domain including environmental, behavioral, and medical [7]. Therefore, it seems reasonable that a further reduction in falls could be brought about when preventative programming has a component of individualization and expands beyond the exercise and educational constructs. What is not known is if there is programming already available beyond the OEP or Stepping On that can add additional value to better achieve a person’s individualized fall risk factors. Therefore, the objective of this scoping review is to examine and synthesize the literature as it relates to programmatic components and clinical outcomes of individualized fall prevention programs on community-dwelling older adults.

## Review

Literature search

Independent searches were performed using PubMed, CINHAL, EMBASE, and PEDro databases. Reproducible search strategies and terms were unique to each database and can be found in Table 1. Search criteria included studies published in English, date range of peer-reviewed publication between January 2011 and May 2021, and included community-based interventions examined via experimental studies meeting the search criteria. Exclusion criteria included if target population <60 years of age, articles with diagnosis-specific criteria (e.g., stroke, Parkinson’s), protocols without population samples, studies that did not have a component of individualized programming, studies where primary outcomes measured were not falls or fall risk, studies where balance interventions were not considered or discussed, and systematic reviews.

**Table 1:** Search terms by database

Database	Search terms	Citations identified
PubMed	“Accidental falls” AND “independent living” AND programs	224
CINAHL	Independent living/community living, older adult/elderly, fall prevention or preventing falls or prevent falls, preventive health care subheadings: accidental fall	49
EMBASE	(“Community living”/exp OR “community living”) AND (“falling”/exp OR falling) AND (“older adults”/exp OR “older adults”)	137
PEDro	Fall and elderly	0

**Figure 1:**
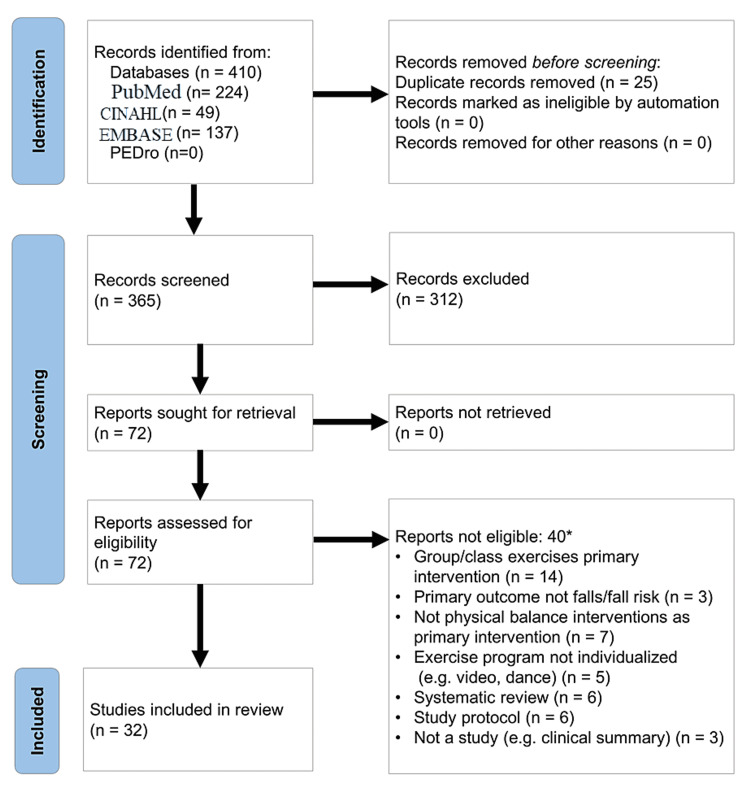
PRISMA flow diagram (identification of studies via databases) *Three studies were excluded for more than one reason. PRISMA: Preferred Reporting Items for Systematic Reviews and Meta-Analyses

Figure 1 details the extraction process for the records obtained [15]. Citations identified were imported into RefWorks (Ann Arbor, MI: ProQuest). The search of the four databases initially yielded a total of 410 citations. Duplicate citations were removed (n=25) yielding 385 citations. Titles and abstracts were assessed for eligibility by pairs of reviewers. Full texts of these potentially eligible studies were then independently evaluated by pairs of reviewers with a third and fourth reviewer providing input into the inclusion decision when there was no consensus, or there was ambiguity. After this level of review, 313 records were excluded. Seventy-two citations remained in second level of eligibility assessment. Using the full manuscript, each was reviewed again to ensure inclusion and exclusion criteria were met, and then extracted data were recorded independently using a standardized data extraction form. Any discrepancies or ambiguities were resolved through discussion. When able, if a program or protocol was previously referenced but not described within the manuscript text, the authors attempted to identify the key program components using the referenced protocol. This review resulted in an additional 39 records being excluded. Thirty-two records remained for final data charting and analysis. A data-charting form was jointly developed by all four reviewers to determine which variables to extract. Each of the four reviewers independently charted the data for all 32 records using a shared online data charting form. Five meetings were conducted to discuss the results, including updating the data-charting form continuously until data was saturated.

Data analysis

The extracted data, which included type of study, program providers, interventions and strategies used to deliver the program, outcomes and measures used, and the statistically significant outcomes of the remaining 32 records were categorized in Tables 2-4 and analyzed [16-48]. Several experimental controlled trials also performed a sub-analysis of in-group improvement for individuals who participated in the intervention groups. These sub-analyses were not included in Table 4 to avoid over-representation of these controlled trials as they were already analyzed experimentally.

**Table 2:** Key screening, assessment, and interventions overview of each program *Controlled experimental trials (randomized or non-randomized). **Two articles both reporting on the HOP-UP-PT program. ***Two articles reporting on the same Otago Exercise and motivational interviewing program. PT: physical therapist; OT: occupational therapist; RN: nurse; CM: case manager; MD: physician; NP: nurse practitioner; psych: psychologist; LPN: license practical nurse; CNA: certified nursing assistant; BP: blood pressure; RA: research assistants; HOP-UP-PT: home-based older persons upstreaming prevention physical therapy

First author, publication year	Program providers	Screening and assessments											Exercise/physical activity interventions				Other interventions/program features					
		Medication (n=15)	BP/orthostatic (n=9)	Cognition (n=21)	Vision (n=16)	Hearing (n=3)	Nutrition (n=7)	Fear of falling/confidence (n=7)	Depression (n=12)	Balance assessment (n=30)	Home assessment (n=16)	Foot assessment (n=3)	Individualized balance training (n=29) OEP (n=12)	Aerobic/endurance exercise (n=13)	Flexibility (n=11)	Strength (n=19)	Motivational interviewing (n=7)	Cognitive training (n=2)	Referral to other providers (n=8)	Specific educational modules (n=15)	Referral to community programs (n=6)	Miscellaneous program features
Johnson et al., 2021 [20]*	PT			x						x			OEP	x		x	x					
Szanton et al., 2021 [21]*	OT, vision specialist, pharmacist	x			x					x	x		x						x			
Arena et al., 2020 [18]**	PT	x	x	x	x		x		x	x	x		OEP	x	x	x	x		x	x	x	Community integration
Davis et al., 2020 [22]*	PT			x					x	x			OEP		x	x						
Levinger et al., 2020 [23]	PT, exercise physiologist	x		x				x	x	x			x		x	x					x	
Pérez-Ros et al., 2020 [24]	PT, RN									x			x		x							
Punlomso et al., 2020 [25]	PT, RN, public health	x	x	x	x				x	x			OEP							x		
Ozic et al., 2020 [26]*	PT, RN	x		x	x	x			x	x	x		x	x								Frailty Measures
Wilson et al., 2020 [19]**	PT	x	x	x	x		x		x	x	x		OEP	x	x	x	x		x	x	x	Community integration
Arkkukangas et al., 2019 [16]*, ***	PT, OT, CM			x						x			OEP	x		x	x			x		
Cederbom and Arkkukangas, 2019 [27]	PT			x				x		x			OEP	x			x				x	
Frith et al., 2019 [28]*	NP, MD	x	x		x					x	x	x	OEP								x	
Kartiko et al., 2019 [29]	PT									x	x		x	x								
Liu-Ambrose et al., 2019 [30]*	PT, MD			x						x			OEP			x						
Mohammed et al., 2019 [31]	not specified		x		x				x	x	x		OEP	x		x			x	x		
Arkkukangas et al., 2018 [17]***	PT			x						x			OEP	x		x	x			x		
Gallo et al., 2018 [32]*	PT							x		x			x		x	x			x			
Suttanon et al., 2018 [33]*	PT	x			x			x		x	x	x	OEP			x				x		
Wetherell et al., 2018 [34]*	PT, psych, orthopedist		x	x	x					x	x		OEP		x	x		x		x	x	
Ciance, 2017 [35]	PT, RN	x		x	x					x	x		x							x		
Otaka et al., 2017 [36]*	PT, MD, OT, physical trainers, RN, dietitian	x		x			x	x	x	x			x	x	x	x				x		
Bamgbade and Dearmon, 2016 [37]	RN, LPN, CNA	x		x	x				x	x	x		x	x					x	x		Incontinence
Ng et al., 2015 [38]*	not specified			x	x	x	x						x			x		x				Frailty Measures
Beauvais and Beauvais, 2014 [39]	RN, students	x	x	x					x	x										x		
Clegg et al., 2014 [40]*	PT			x	x				x	x						x					x	
Cohen et al., 2015 [41]*	RN									x	x		x									
Moller et al., 2014 [42]*	PT, RN	x					x			x	x		x			x			x	x	x	
Palvanen et al., 2014 [43]*	PT, RN, MD	x	x	x	x		x	x	x	x	x		x	x	x	x				x		
Luck et al., 2013 [44]*	RN, psych, sociologist			x	x		x				x								x			Multi-disciplinary Team Meeting
Pérula et al., 2012 [45]*	PT, MD, RN	x	x	x	x	x		x		x	x	x	x	x	x	x	x			x		
Robitaille et al., 2012 [46]	PT, rehabilitation technicians and educators									x			x			x				x		
Jacobson et al., 2011 [47]*	RA									x			x		x							

**Table 3:** Frequency of evidenced-based outcomes measured (outcomes measured and frequency) *Two articles reporting on the same program. **Short physical performance battery includes five times sit to stand, gait speed over 3 or 4 meters, and four-stage balance test. ***Authors indicated test was modified. ****Only trail making part B assessed. UCLA: University of California Los Angeles; STEADI: stopping elderly accidents deaths and injuries

Topic	Specific assessment measure	References	Number of references
Health and medical screenings (n=27)*	Body mass index (BMI)	[18,19,24,31,36,38]	6
	Blood pressure	[18,19,24,31,43]	5^*^
	Functional comorbidity index	[18,19,22,30,40]	5
	Vision screen	[33,35,43]	3
	Orthostatic hypotension	[18,19,43]	3^*^
	Charlson Comorbidity Index	[40]	1
	Body fat percentage	[24]	1
	Calcaneal speed of sound (bone density)	[36]	1
	Pain	[36]	1
	Reaction time	[43]	1
	Tilburg Frailty Indicator (TFI)	[26]	1
	Ng frailty assessment	[38]	1
Balance (n=23)*	Berg Balance Scale	[25,32,42,47]	4
	Step test or step-up test	[23,33,47]	3
	Functional reach	[33,35,36]	3
	4-Stage Balance Test	[18,19,28]	3^*^
	Tinetti performance-oriented mobility assessment test	[35,45]	2
	Mini-BESTest	[16,20]	2
	One-legged stance test	[36,46]	2
	Tandem stance	[21,46]	2
	Fukuda stepping test	[42]	1
	Tandem walk test	[46]	1
	Otago exercise level	[18,19]	2^*^
Physical function (21)*	Timed up and go test (TUG)	[18,19,21,22,28-33,35,36,10,42,43]	15^*^
	Short physical performance battery (SPPB)**	[16,20,22,27,30,43]	6
	8-foot up and go test	[47]	1
Falls (n=17)	Fall frequency	[22,24,30,32-34,36,37,41,43-46]	13
	Fall-related fractures or injury	[30,41,43,45]	4
Falls efficacy/confidence (n=15)*	Fall Efficacy Scale-International (FES-I)	[34,35,39]	3
	Modified Falls Efficacy Scale	[18,19,24,33]	4^*^
	Fall Efficacy Scale Swedish version (FES{S})	[16,20]	2
	Fear of falling	[23,36]	2
	Tinetti Fall Efficacy Scale	[21,24]	2
	General Falls Efficacy Scale	[17]	1
	Confidence scale when performing activities**	[29]	1
	Activities balance confidence scale	[32]	1
Strength (including functional strength) (n=15)*	Grip strength measured with handgrip dynamometer	[16,20,24,36,43]	5
	30-second chair stand test	[23,28,31,47]	4
	Five Times Sit to Stand (5×STS)	[18,19,32,33]	4^*^
	Quadricep strength measured with dynamometer	[25,38,43]	3
Cognition (n=14) *	Mini Mental Status Exam (MMSE)	[22,27,30,36,40,42]	6
	Trail making part A and B	[18,19,22,30]	4*^,^****
	Mini-cog	[18,19,39]	3^*^
	Montreal cognitive assessment	[22,30]	2
	Stroop color word test, digit symbol substitution test	[30]	1
Physical activity (n=11)*	Exercise adherence diary	[16,17,20,37]	4^*^
	Frändin/Grimby activity score	[16,17,20]	3^*^
	Physical Activity Scale for the Elderly (PASE)	[30,33]	2
	Frenchay Activity Index	[36]	1
	Weekly hours of exercise in previous 12-months (self-report)	[24]	1
	Self-efficacy for exercise	[23]	1
	Physical Activity Enjoyment Scale (PACES)	[23]	1
Extrinsic risk factors (n=10)*	Home environment	[18,19,31,33,37,42,43]	7^*^
	General footwear assessment	[33,35]	2
	Health Behavior Questionnaire	[18,19]	2*
	Mini Nutritional Assessment Short Form (MNA-SF)	[24]	1
Wellbeing, psychosocial health, and depression (8)*	Geriatric Depression Scale (GDS)-original or short	[22,23,30,36,40]	5
	Patient Health Questionnaire (PHQ)-9	[18,19]	2^*^
	UCLA 3-item loneliness scale	[23]	1
	WHO 5 Wellbeing Questionnaire	[23]	1
Fall risk (n=7)*	STEADI Questions	[18,19,28,29]	4^*^
	Algorithm for objective fall risk	[34]	1
	Downton Fall Risk Index	[42]	1
	The Falls Risk for Older People in the Community (FROP-Com)	[23]	1
	CAREFALL Triage Instrument (CTI)	[31]	1
Activities of daily living (n=7)	Barthel Index	[24,44]	2
	Modified Barthel Index	[40]	1
	Lawton Instrumental Activities of Daily Living Scale	[44]	1
	Groningen Activity Restriction Scale	[26]	1
	Activities of daily living (ADL) staircase	[42]	1
	General Motor Function Assessment Scale	[42]	1
Quality of life and global health (n=6)	EuroQoL Group 5-Dimension (EQ-5D)	[23,40]	2
	Euro-QoL 5D 3-Level Quality of Life Scale (EQ-5D-3L)	[22,27]	2
	Short form 6D (health status)	[22]	1
	15D health-related quality of life instrument	[39]	1
Gait speed (n=2)	4-meter Walk Test	[23]	1
	6-meter fast gait speed test	[38]	1
Endurance (n= 1)	2-minute walk test	[23]	1

**Table 4:** Frequency of statically significant (P< 0.05) improvement in falls and falls risks measures *Fear of falling improved for those who were most fearful at baseline measure. ABC: activities-specific balance confidence scale; FES: Falls Efficacy Scale; CTS test: chair to stand test; MMT: manual muscle test; TUG: timed up and go test; SPPB: short physical performance battery

Change in falls or fall risk measures	Controlled trials		Quasi-experimental	
	Statistically significant improvement	No statistical improvement	Statistically significant improvement	No statistical improvement
Frequency of falls	n=8 [22,28,30,36,41,43-45]	n=5 [20,32-34,42]	n=4 [23,24,31,37]	n=1 [46]
Fall injury	n=2 [40,42]	n=2 [29,44]	-	-
Fear of falling or fall efficacy (includes fear of falling, ABC, FES)	n=4 [21,32,34,45]	n=2 [16,33]	n=3 [23,29,39]*	n=1 [17]
Balance (includes Berg, Tinetti, four-stage balance test, Tandem stance, Romberg, functional reach, step test)	n=6 [21,28,32,36,45,47]	n=5 [16,20,30,33,42]	n=5 [18,23,25,31,46]	-
Strength including functional strength (includes dynamometry, chair rise 5×, 30 second CTS test, MMT)	n=3 [32,36,47]	n=4 [16,20,33,38]	n=4 [23-25,31]	n=1 [18]

Program providers were descriptively analyzed. Frequency counts were used for the various interventions and strategies used for program delivery, outcome tools and measures used, and for the statistically significant outcomes reported. Key measures were examined for effectiveness when statistically significant improvement was reported in both controlled trials and observational studies. Correlations are presented as percent differences in Table 5. 

**Table 5:** Interventions provided in relation to statistically significant (P< 0.05) improvements for controlled trials (n=19)

Total number of controlled trials (n=19)		Statistical improvement in falls (n=8) percent (frequency)	No statistical improvement in falls (n=8) percent (frequency)	Percent difference	Statistical improvement in balance (n=6) percent (frequency)	No statistical improvement in balance (n=5) percent (frequency)	Percent difference
Screening and assessments	Medication	50% (4)	40% (2)	10	66.7% (4)	40% (2)	26.7
	BP/orthostatic	37.5% (3)	20% (1)	17.5	33.3% (2)	0% (0)	33.3
	Cognition	75% (6)	40% (2)	35	33.3% (2)	60% (3)	-26.7
	Vision	50% (4)	40% (2)	10	50% (3)	20% (2)	30
	Hearing	12.5% (1)	0% (0)	12.5	16.7% (1)	0% (0)	16.7
	Nutrition	37.5% (3)	20% (1)	17.5	16.7% (1)	20% (1)	-3.3
	Fear of falling	37.5% (3)	40% (2)	2.5	50% (3)	20% (1)	30
	Depression	37.5% (3)	0% (0)	37.5	16.7% (1)	0% (0)	16.7
	Balance assessment	87.5% (7)	100% (5)	-12.5	100% (6)	100% (5)	0
	Home assessment	62.5% (5)	60% (3)	2.5	66.7% (3)	40% (2)	26.7
	Foot assessment	25% (2)	20% (3)	5	33.3% (2)	20% (1)	13.3
Exercise/Physical activity interventions	Individualized Balance Training	87.5% (7)	100% (3)	-12.5	83.3% (5)	100%	-16.7
	Aerobic/endurance exercise	37.5% (3)	20% (1)	17.5	33.3% (2)	40% (2)	-6.7
	Flexibility	50% (4)	40% (2)	10	66.7% (4)	0% (0)	66.7
	Strength	62.5% (5)	100% (5)	-37.5	50% (3)	100% (5)	-50
	Motivational interviewing	12.5% (1)	20% (1)	-7.5	16.7% (1)	40% (2)	-23.3
Other interventions/program features	Cognitive training	0% (0)	20% (1)	-20	0% (0)	0% (0)	0
	Referral to other providers	12.5% (1)	40% (2)	-27.5	16.7% (1)	20% (1)	0
	Specific educational modules	37.5% (3)	60% (3)	-22.5	16.7% (1)	60% (3)	-3.3
	Referral to community programs	12.5% (1)	40% (2)	-27.5	16.7% (1)	40% (2)	-23.3

Clinical implications

Findings from our scoping review revealed that health care providers, such as PTs, have a substantial role in the creation, administration, and assessment of individualized fall prevention programming. Specifically, rehabilitation professionals (PTs and OTs), nurses, and physicians are among the healthcare disciplines most utilized when providing fall reduction programs. However, the significant role of the PT in designing and administering these programs was notable, considering that more than half of the programs utilized PTs as a member of the care team, and about one-third utilized them as the sole healthcare provider. As PTs are movement specialists who provide individualized and multisystem body assessments to deliver targeted interventions and education, they can serve as a bridge between community programming and the healthcare system. PTs are also well equipped to provide their services in a variety of settings including the home, physician offices, community centers, and rehabilitation clinics which optimizes their positioning to address fall prevention across the continuum of care.

It should also be noted that the OEP was utilized and studied at an increased frequency in more recently published studies. The OEP includes several components to address fall risk factors including balance, strength, flexibility, and aerobic walking exercise. While our scoping review was unable to fully determine if all aspects were performed in the studies analyzed, there is some evidence that many of the OEP components are successful in reducing fall rate and risk. As the OEP is further investigated and supported, it may provide a criterion standard for exercise programming, serving as one component of a multimodal fall prevention program.

Our study identified a lack of congruency in the outcome measurements utilized to assess fall risk factors and therefore, it is difficult to extrapolate result heterogeneity. The two most frequently used measures were fall frequency and the timed up and go (TUG), with each appearing internationally and demonstrating strong constructs for reproducibility (Table 3) [49,50]. While fall frequency can provide metrics on incidence and efficacy of fall prevention programming, a physical measure such as the TUG can provide insight on fall risk factors when considering components of reaction time, strength, gait speed, balance, safety, and overall functional mobility. It was noted in this scoping review of the literature that the TUG and SPPB did not consistently demonstrate improvements in several studies. As these tests measure multiple domains of movement, they appear to be a useful proxy measure of global function and therefore, would have utility in a multifactorial screening. However, as there was substantial variability in interventions, positive outcomes may have been limited by the interventions provided as opposed to the outcome measure chosen.

Balance, physical functioning, falls, fall efficacy, strength, and various medical screenings/assessments were the most common outcomes measured in the studies included in this review (Table 4). Other areas applicable to fall risk were less frequently identified among the articles reviewed. These included cognition, physical activity levels, extrinsic factors (e.g., home environment), and psychosocial health (e.g., depression). Although less frequently used, there is some evidence for their utility in providing a multifactorial program that best suits a person’s unique needs [51,52]. Specifically, cognition was identified as a key measure that was tested more often in controlled trials that improved falls and balance. This supposition is supported in the diverging outcomes among the controlled trials’ key study results. This highlights the need for continued research to determine what program characteristics are best administered to address each older adult's unique fall risk needs and circumstances.

With the review of controlled trials in Table 5, there is increased emphasis on the importance of assessing and intervening in multiple domains of health and functioning. The psychosocial well-being of older adults can play a significant role in fall reduction and prevention [51,52]. The current review also indicates that the key components to improve balance as a fall risk factor may include orthostatic hypotension assessments and the addition of flexibility training in fall-prevention programming.

Factors that were less impactful in improving balance were strengthening exercise, referral to other providers/community programs, and specific education modules. The heterogeneity of strength measures reported made it difficult to determine the impact of isolated muscle strength, as measured through dynamometry, compared to functional strength and motor control. Furthermore, we did not find consistent evidence of the role of strength exercises to improve balance; conversely, Lee and Park, and Eckardt noted improvements in balance in older adults following a strengthening program [53,54].

Limitations

A systematic review and/or meta-analysis would provide more inference to the combined results of the included studies versus our scoping review of the literature. Furthermore, omission, exclusion, or inconsistent reporting of program protocols may have resulted in incomplete or limited description of specific interventions delivered. For example, while several studies reported the use of the OEP, it was unclear if all OEP components were performed at the recommended dosing or if modifications were introduced (e.g., self-guided walking program).

Future research

Future research is warranted to investigate cost savings associated with individualized programs for those at risk of fall. Specifically, large-scale cost analyses of varied intervention strategies would be important. Current evidence demonstrates that the cost of multifactorial fall prevention programs depends significantly on the age of participants and the decision makers’ willingness to pay to prevent a fall [55]. Our review did not include studies that were primarily focused on telehealth or group programming. While previous research has supported the efficacy of these elements, a more specific assessment of their ability to provide effective clinical outcomes would improve the current state of research that lacks clear standards of care.

## Conclusions

This study provides a summary of key features and clinical outcomes associated with individualized fall prevention programs for community-dwelling older adults. While many programs reduced fall frequency and improved balance of older adults, there was no conclusive evidence as to which assessments and interventions were optimal to deliver as individualized fall prevention programming. Additionally, a lack of congruence among the program outcome measures limited the ability to identify which assessments were most useful in quantifying fall risk domains. However, there is evidence that the skill of a PT and measures of fall frequency, balance, and function are common. Despite the variability among programs, there is emerging evidence that individualized, multimodal fall prevention programs may improve fall risk of community-dwelling older adults and convenient access to these programs should be emphasized.
